# Draft Genome Sequence of *Mycobacterium austroafricanum* DSM 44191

**DOI:** 10.1128/genomeA.00317-14

**Published:** 2014-04-17

**Authors:** Olivier Croce, Catherine Robert, Didier Raoult, Michel Drancourt

**Affiliations:** Aix Marseille Université, URMITE, Marseille, France

## Abstract

We announce the draft genome sequence of *Mycobacterium austroafricanum* DSM 44191^T^ (= E9789-SA12441^T^), a non-tuberculosis species responsible for opportunistic infection. The genome described here has a size of 6,772,357 bp with a G+C content of 66.79% and contains 6,419 protein-coding genes and 112 RNA genes.

## GENOME ANNOUNCEMENT

*Mycobacterium austroafricanum* was named after the initial isolation of a set of 23 strains from water in South Africa (1). Numerical taxonomy indicated that these isolates are representative of a new species related to *Mycobacterium parafortuitum* (1). Further genetic analyses indicated that, in fact, *M. austroafricanum* belongs to the *Mycobacterium vaccae* complex, which also contains *Mycobacterium vanbaalenii*, and is more distantly related to *Mycobacterium aurum* and *Mycobacterium pyrenivorans* (2). *M. austroafricanum* is an environmental organism isolated from soil, in particular, from hydrocarbon-polluted soils (3, 4). Indeed, *M. austroafricanum* has attracted much attention because it is able to degrade gasoline hydrocarbons (5, 6). *M. austroafricanum* has rarely been isolated from patients (7, 8), and while *M. austroafricanum* DNA has been detected in diseased joint fluids, the clinical significance of *M. austroafricanum* has not yet been established (9).

We therefore sequenced the whole genome of the *M. austroafricanum* DSM 44191 (E9789-SA12241^T^) strain in order to illustrate its phylogenetic relationship with closely related mycobacteria and to help depict its unique metabolic capabilities.

Genomic DNA was isolated from *M. austroafricanum* grown in Middlebrook 7H9 broth (Becton Dickinson, Sparks, MD) at 37°C. It was then sequenced using three high throughput NGS technologies: Roche 454 (Roche Diagnostics Corporation, Indianapolis, IN) (10), SOLiD version 4 (Life Technologies, Carlsbad, CA), and MiSeq Illumina (Illumina Inc., San Diego, CA). A 3.74-kb paired-end library was loaded on a picotiter plate and sequenced with the Roche-GS FLX Titanium Sequencing Kit XLR70. The run yielded 143.9 Mb with 435,968 passed filters and an average length of 329 bp. The bar-coded paired-end SOLiD library generated 975,705 reads of 50× 35-bp length. Finally, a paired-end Nextera library, fragmented at 800 bp, sequenced on MiSeq at 2× 151 bp, yielded 823,878 reads with an indexing of 6.57% on the flowcell.

Reads from these various sequencing technologies were first assembled separately. The 454 reads were then assembled into contigs and scaffolds using Newbler version 2.8 (Roche). Illumina reads were trimmed using Trimmomatic (11), then assembled using the Spades software (12, 13). The obtained contigs were combined by SSPACE (14) and Opera software v1.2 (15) complemented by GapFiller v1.10 (16). The genome was improved using CLC Genomics v5 software (CLC bio, Aarhus, Denmark).

The *M. austroafricanum* draft genome sequence consists of 20 scaffolds of 69 contigs containing 6,682,536 bp, with an estimated genome size including gaps of 6,772,357 bp. The G+C content of this genome is 66.79%. Noncoding genes and miscellaneous features were predicted using RNAmmer (17), ARAGORN (18), Rfam (19), and PFAM (20). Open reading frames were predicted using Prodigal (21), and functional annotation was achieved using BLASTp against the GenBank database (22) and the Clusters of Orthologous Groups (COG) database (23, 24). The genome was shown to encode at least 112 predicted RNAs, including 4 rRNAs, 85 tRNAs, 2 transfer-messenger RNAs, and 21 miscellaneous RNAs. Also, 6,419 genes were identified, which yields a coding capacity of 6,124,875 bp (coding percentage, 90.4%). Among these genes, 850 (12.77%) were found to encode putative proteins and 1,324 (16.88%) were assigned as encoding hypothetical proteins. Moreover, 6,549 genes matched at least one sequence in the COG database with BLASTp default parameters.

### Nucleotide sequence accession numbers.

The *Mycobacterium austroafricanum* strain DSM 44191 (= E9789-SA12441^T^) genome sequence has been deposited at DDBJ/EMBL/GenBank under the accession no. HG964450 to HG964469. The whole-genome shotgun master numbers are CCAW010000001 to CCAW010000069.

## References

[1] Tsukamura M Van der Meulen HJ Grabow WOK 1983 Numerical taxonomy of rapidly growing, scotochromogenic mycobacteria of the *Mycobacterium parafortuitum* complex: *Mycobacterium austroafricanum* sp. nov. and *Mycobacterium diernhoferi* sp. nov., nom. rev. Int. J. Syst. Evol. Microbiol. 33:460–469. 10.1099/00207713-33-3-460

[2] Derz K Klinner U Schuphan I Stackebrandt E Kroppenstedt RM 2004 *Mycobacterium pyrenivorans* sp. nov., a novel polycyclic-aromatic-hydrocarbon-degrading species. Int. J. Syst. Evol. Microbiol. 54:2313–2317. 10.1099/ijs.0.03003-0 15545477

[3] Leys NM Ryngaert A Bastiaens L Wattiau P Top EM Verstraete W Springael D 2005 Occurrence and community composition of fast-growing *Mycobacterium* in soils contaminated with polycyclic aromatic hydrocarbons. FEMS Microbiol. Ecol. 51:375–388. 10.1016/j.femsec.2004.09.015 16329885

[4] Wang Y Ogawa M Fukuda K Miyamoto H Taniguchi H 2006 Isolation and identification of mycobacteria from soils at an illegal dumping site and landfills in Japan. Microbiol. Immunol. 50:513–524. 10.1111/j.1348-0421.2006.tb03821.x 16858142

[5] Maciel H Mathis H Lopes Ferreira N Lyew D Guiot S Monot F Greer CW Fayolle-Guichard F 2008 Use of *Mycobacterium austroafricanum* IFP 2012 in a MTBE-degrading bioreactor. J. Mol. Microbiol. Biotechnol. 15:190–198. 10.1159/000121330 18685271

[6] House AJ Hyman MR 2010 Effects of gasoline components on MTBE and TBA cometabolism by *Mycobacterium austroafricanum* JOB5. Biodegradation 21:525–541. 10.1007/s10532-009-9321-8 20012341

[7] Singh S Gopinath K Shahdad S Kaur M Singh B Sharma P 2007 Nontuberculous mycobacterial infections in Indian AIDS patients detected by a novel set of ESAT-6 polymerase chain reaction primers. Jpn. J. Infect. Dis. 60:14–18 17314419

[8] Shojaei H Heidarieh P Hashemi A Feizabadi MM Daei Naser A 2011 Species identification of neglected nontuberculous mycobacteria in a developing country. Jpn. J. Infect. Dis. 64:265–271 21788699

[9] Van der Heijden IM Wilbrink B Schouls LM van Embden JD Breedveld FC Tak PP 1999 Detection of mycobacteria in joint samples from patients with arthritis using a genus-specific polymerase chain reaction and sequence analysis. Rheumatology (Oxford) 38:547–553. 10.1093/rheumatology/38.6.547 10402076

[10] Margulies M Egholm M Altman WE Attiya S Bader JS Bemben LA Berka J Braverman MS Chen Y-J Chen Z Dewell SB Du L Fierro JM Gomes XV Godwin BC He W Helgesen S Ho CH Irzyk GP Jando SC Alenquer MLI Jarvie TP Jirage KB Kim J-B Knight JR Lanza JR Leamon JH Lefkowitz SM Lei M Li J Lohman KL Lu H Makhijani VB McDade KE McKenna MP Myers EW Nickerson E Nobile JR Plant R Puc BP Ronan MT Roth GT Sarkis GJ Simons JF Simpson JW Srinivasan M Tartaro KR Tomasz A Vogt KA Volkmer GA Wang SH Wang Y Weiner MP Yu P Begley RF Rothberg JM 2005 Genome sequencing in microfabricated high-density picolitre reactors. Nature 437:376–380. 10.1038/nature03959 16056220 PMC1464427

[11] Lohse M Bolger AM Nagel A Fernie AR Lunn JE Stitt M Usadel B 2012 RobiNA: a user-friendly, integrated software solution for RNA-Seq-based transcriptomics. Nucleic Acids Res. 40:W622–W627. 10.1093/nar/gks540 22684630 PMC3394330

[12] Nurk S Bankevich A Antipov D Gurevich AA Korobeynikov A Lapidus A Prjibelski AD Pyshkin A Sirotkin A Sirotkin Y Stepanauskas R Clingenpeel SR Woyke T McLean JS Lasken R Tesler G Alekseyev MA Pevzner PA 2013 Assembling single-cell genomes and mini-metagenomes from chimeric MDA products. J. Comput. Biol. 20:714–737. 10.1089/cmb.2013.0084 24093227 PMC3791033

[13] Bankevich A Nurk S Antipov D Gurevich AA Dvorkin M Kulikov AS Lesin VM Nikolenko SI Pham S Prjibelski AD Pyshkin AV Sirotkin AV Vyahhi N Tesler G Alekseyev MA Pevzner PA 2012 SPAdes: a new genome assembly algorithm and its applications to single-cell sequencing. J. Comput. Biol. 19:455–477. 10.1089/cmb.2012.0021 22506599 PMC3342519

[14] Boetzer M Henkel CV Jansen HJ Butler D Pirovano W 2011 Scaffolding pre-assembled contigs using SSPACE. Bioinformatics 27:578–579. 10.1093/bioinformatics/btq683 21149342

[15] Gao S Sung WK Nagarajan N 2011 Opera: reconstructing optimal genomic scaffolds with high-throughput paired-end sequences. J. Comput. Biol. 18:1681–1691. 10.1089/cmb.2011.0170 21929371 PMC3216105

[16] Boetzer M Pirovano W 2012 Toward almost closed genomes with GapFiller. Genome Biol. 13:R56. 10.1186/gb-2012-13-6-r56 22731987 PMC3446322

[17] Lagesen K Hallin P Rødland EA Staerfeldt HH Rognes T Ussery DW 2007 RNAmmer: consistent and rapid annotation of ribosomal RNA genes. Nucleic Acids Res. 35:3100–3108. 10.1093/nar/gkm160 17452365 PMC1888812

[18] Laslett D Canback B 2004 ARAGORN, a program to detect tRNA genes and tmRNA genes in nucleotide sequences. Nucleic Acids Res. 32:11–16. 10.1093/nar/gkh152 14704338 PMC373265

[19] Griffiths-Jones S Bateman A Marshall M Khanna A Eddy SR 2003 Rfam: an RNA family database. Nucleic Acids Res. 31:439–441. 10.1093/nar/gkg006 12520045 PMC165453

[20] Punta M Coggill PC Eberhardt RY Mistry J Tate J Boursnell C Pang N Forslund K Ceric G Clements J Heger A Holm L Sonnhammer EL Eddy SR Bateman A Finn RD 2012 The Pfam protein families database. Nucleic Acids Res. 40:D290–D301. 10.1093/nar/gkr1065 22127870 PMC3245129

[21] Hyatt D Chen GL Locascio PF Land ML Larimer FW Hauser LJ 2010 Prodigal: prokaryotic gene recognition and translation initiation site identification. BMC Bioinformatics 11:119. 10.1186/1471-2105-11-119 20211023 PMC2848648

[22] Benson DA Karsch-Mizrachi I Clark K Lipman DJ Ostell J Sayers EW 2012 GenBank. Nucleic Acids Res. 40:D48–D53. 10.1093/nar/gkr1202 22144687 PMC3245039

[23] Tatusov RL Galperin MY Natale DA Koonin EV 2000 The COG database: a tool for genome-scale analysis of protein functions and evolution. Nucleic Acids Res. 28:33–36. 10.1093/nar/28.1.33 10592175 PMC102395

[24] Tatusov RL Koonin EV Lipman DJ 1997 A genomic perspective on protein families. Science 278:631–637. 10.1126/science.278.5338.631 9381173

